# Special Interests in Autism: Functions, Benefits, and Challenges

**DOI:** 10.1177/13623613261471572

**Published:** 2026-08-06

**Authors:** Mareike Bayer, Isabel Dziobek

**Affiliations:** 1 Humboldt-Universität zu Berlin, Germany; 2 German Center for Mental Health (DZPG), partner site Berlin, Germany

**Keywords:** special interests, emotion regulation, resources, self, stigma

## Abstract

Special interests are a highly prevalent feature of autism spectrum conditions (ASC) and have long been described primarily from a deficit-oriented perspective. More recent work has pointed to their potential as psychological resources, yet systematic evidence and direct comparisons with non-autistic groups remain limited. The present study examined the significance of special interests for well-being, self-related aspects and emotion regulation in autistic adults, as well as negative consequences and stigma. Furthermore, we directly compared the likelihood of engaging in special interests versus seeking social contact across a range of emotional contexts. A total of 182 participants (60 autistic and 122 non-autistic) completed an online survey on multiple aspects of their (special) interests and measures of social anxiety and self-esteem. Autistic individuals reported a stronger role of special interests in emotion regulation and as a source of learning and knowledge, while also experiencing greater stigma and negative consequences compared with non-autistic participants. Autistic participants further showed a higher likelihood of relying on their interests as a coping strategy in negative and exhausting situations, highlighting their particular relevance for emotion regulation. Our findings emphasize the unique functions of special interests in autism and point to their therapeutic potential as adaptive resources.

**Lay Abstract:**

Many autistic people have special interests – topics or activities they pursue with great passion. We asked autistic and non-autistic adults how meaningful their interests are and how they use them in different emotional situations. Autistic people reported that interests are especially helpful for coping with stress and regulating emotions, and that they are an important source of learning and knowledge. On the other hand, autistic individuals also experienced more stigma and negative reactions from others than non-autistic individuals. Our findings show that special interests are an important source of well-being and should be recognized as a valuable resource in autism.

## Introduction

Special interests are a part of the diagnostic domain of restricted, repetitive patterns of behavior, interests and activities (American Psychiatric Association, 2013) of Autism Spectrum Conditions (ASC). They are characterized by an intense preoccupation with a circumscribed range of subjects and have a high prevalence in ASC of around 75% to 95% throughout childhood, adolescence and adulthood (Lam et al., 2008; Nowell et al., 2021; Turner-Brown et al., 2011). For a long time, researchers and clinicians have employed a deficit-oriented view on special interests, focusing primarily on potential negative consequences of special interests for social functioning (Klin et al., 2007), or postulating that special interests might interfere with social interactions and might cause difficulties in parent–child interactions (Turner-Brown et al., 2011). This may in part reflect the focus of many parent-report studies on potential challenges and functional impairment associated with intense interests (Klin et al., 2007; Turner-Brown et al., 2011). In contrast, autistic individuals’ perspectives on special interests, as well as parents’ views on their positive aspects, have rarely been investigated. This negative stance is also evident in the nomenclature employed to describe these interests as “circumscribed” or “restricted,” even though more recent evidence suggests that special interests extend across a wide range of topics and that individuals often report several interests concurrently (Nowell et al., 2021).

More recently, however, there has been a broader shift in research on special interests toward a stronger focus on lived experience and positive aspects, reflected both in increased attention to autistic people’s self-reports and to strength-based parent perspectives. In this context, Mercier and colleagues (2000) interviewed autistic individuals and their relatives, and were among the first to portray special interests as a potential source of well-being, personal validation and personal growth for autistic individuals. Furthermore, reports from autistic children and youth as well as their parents suggested that engagement in special interests was associated not only with a positive self-image and self-confidence, but also with increases in social interactions (Winter-Messiers, 2007). More recent work has further emphasized that specific interests may support psychological well-being (Cooper et al., 2024; Nowell et al., 2021), foster communication and social connection (Long, 2025), and may also provide a sense of safety and protection during social interactions (Lizon et al., 2024). It is therefore not surprising that the vast majority of autistic adults view their special interest as a strength that should be encouraged (Koenig & Williams, 2017). In line with the shift away from deficit-oriented accounts, more recent parent-report work has similarly emphasized strengths and positive qualities in autistic children and young people, and has also highlighted the value of strengths- and interest-based approaches (Cost et al., 2021; Lee et al., 2020; Lei et al., 2026; Warren et al., 2021; Wilkinson et al., 2022).

In an attempt to understand the motivation to engage with special interests, Grove and colleagues (2016) developed the “special interest motivation scale” and showed that autistic individuals reported higher intrinsic motivation, as well as engagement and flow, compared with a non-autistic group as reasons for engaging in their interests. Furthermore, the degree of intrinsic motivation was related to higher quality of life (Grove et al., 2018). Finally, special interests were identified as a source of expertise that can potentially be useful for finding employment (Kirchner & Dziobek, 2014).

From a mental health perspective, a further important aspect is the potential role of special interests in emotion regulation. Alterations in emotion regulation are common in ASC, and previous research has suggested that autistic individuals may show different patterns of emotion regulation than non-autistic individuals, including reduced flexibility across contexts (Cai et al., 2018). Several studies reported that autistic individuals used their special interests to cope with negative feelings, to calm down (Teti et al., 2016), to shield themselves from stressful situations (Lizon et al., 2024), and to reduce their general anxiety (Kaboski et al., 2015; Koenig & Williams, 2017). Furthermore, special interests have been suggested as a potential protective resource against autistic burnout (Mantzalas et al., 2022). These findings are highly relevant since ASC is associated with high rates of co-occurring anxiety and depression of around 40% to 60% (Strunz et al., 2014; Tebartz van Elst et al., 2013), thus warranting new strategies that draw on individual autistic resources to reduce psychological distress and support mental health.

In the present study, we examined the potential of special interests to serve as psychological resources in the lives of autistic adults, aiming to provide a more nuanced understanding of their significance. While previous research has already addressed certain aspects of challenges and strengths associated with special interests, there is still limited insight regarding the broader role of special interests for individual well-being. Moreover, many previous studies did not include non-autistic control groups, even though engaging in interests (or hobbies, as they are typically referred to in this context) is also highly common among non-autistic individuals. However, such engagement is likely characterized by a wider variation in intensity, which is why we also assessed the amount of time participants devote to their interests. Relevant aspects of special interests were identified based on earlier findings (e.g., Grove et al., 2018; Harrop et al., 2019; Winter-Messiers, 2007) and input from autistic experts by experience, and included the domains Self-worth, Emotion Regulation, Source of Positive Emotions, Source of Learning and Knowledge, and Social Identity. In addition, we assessed negative consequences and stigma associated with special interests.

A second aim of the study was to explore the role of special interests as a strategy for emotion regulation, which had been suggested by previous research (e.g., Koenig & Williams, 2017). Crucially, we considerably broadened the relevant research focus: First, we not only assessed the likelihood of engaging in special interests as a means of emotion regulation in negative and exhausting situations but also included a range of positive situations for comparison. Second, we directly compared the likelihood of engaging in special interests with the likelihood of seeking social contact or support. Third, including a non-autistic control group allowed us to examine whether and how the role of special interests might differ between groups. Finally, by including measures of social anxiety and self-esteem, we explored potential links between engagement in special interests and mental health.

We predicted a high significance of special interests in ASC, as evident in higher scores on all subscales (Self-worth, Emotion Regulation, Source of Positive Emotions, Source of Learning and Knowledge, Social Identity, and Negative Consequences and Stigma) for autistic compared with non-autistic individuals, as well as a higher amount of time spent engaging in special interests. Regarding the likelihood to engage in special interests compared with social contact, we expected that across emotional contexts, autistic individuals would show a higher tendency to engage in special interests and a lower tendency for social strategies compared with the control group.

## Method

### Measures

#### Survey Development

##### Part 1 – Positive Aspects and Stigma

In Part 1 of the survey, we investigated the positive aspects of special interests^1^ as well as negative consequences for the individual. The selection of positive subdomains was informed by previous literature indicating that special interests can be linked to well-being, enjoyment, engagement, and social connection in autistic people, and may also function as strengths that support learning and participation (Grove et al., 2018; Harrop et al., 2019; Winter-Messiers, 2007), as well as by input from autistic experts by experience from the Autism Research Cooperation (Autismus-Forschungs-Kooperation; AFK).^2^ Domain selection and item development followed an iterative process within the research team and with feedback from an expert by experience regarding clarity and relevance.

Subdomains included *Source of Positive Emotions* (special interests as a source of joy, inspiration, excitement and flow), *Self-worth* (feeling content, proud, or confident as a result of engaging in special interests), *Source of Learning and Knowledge* (special interests as a possibility to acquire facts and knowledge), *Emotion Regulation* (special interests as a means to cope with difficult or stressful situations), and *Social Identity* (connecting with others through special interests). In contrast to the positive subdomains, the Negative Aspects and Stigma scale was conceived more broadly to capture adverse experiences such as conflict, negative social reactions, stigmatization, and other burdens associated with engagement in interests. It should therefore be understood as an additional aspect of special interests rather than a dimension directly parallel to the positive subdomains. The survey included 43 items in total (six to nine questions per subscale). Answers were provided on a 7-point Likert-type scale ranging from “not at all” to “completely”; in addition, participants had the option to indicate that they were not able to assess this item (e.g., if they did not have a concept of a certain emotional term used in the question).

##### Part 2 – Emotion Regulation

In the second part of the survey, we asked our participants to indicate the likelihood of engaging with their special interests and of seeking social contact in a variety of negative, positive, and exhausting or stressful situations (19 in total), for example, “When I received bad news, . . . a) I am likely to engage in my special interest” b) “I am likely to seek contact to a familiar person.” The 19 situations were developed through an iterative process within the research team and with autistic experts by experience. The aim was to generate situations that represented a range of emotional contexts in which different regulatory responses might be relevant. Participants provided the likelihood for both strategies (social and interest) on a 7-point Likert-type scale ranging from “not at all likely” to “highly likely.” In addition, they had the opportunity to indicate other reactions if they considered them relevant. Situations were grouped into three categories: positive situations (e.g., feeling content, receiving good news), negative situations (e.g., feeling anxious, sad, or lonely), and states of exhaustion (e.g., feeling tired or irritated).

#### Additional Questionnaires

In addition, participants completed three established self-report questionnaires: Autistic traits were assessed using the Autism Spectrum Quotient (Baron-Cohen et al., 2001), a 50-item measure for autistic traits in adults with established reliability and validity (Hoekstra et al., 2008). Social anxiety was assessed using the Liebowitz Social Anxiety Scale (LSAS; Liebowitz, 1987), which measures anxiety and avoidance with good psychometric properties (Fresco et al., 2001); anxiety and avoidance ratings were combined into a total score. Global self-esteem was assessed using the Rosenberg Self-Esteem Scale (Rosenberg, 1965; for psychometric properties, see Sinclair et al., 2010), a 10-item measure of overall perceived self-worth.

### Participants

Our sample consisted of adult individuals (at least 18 years of age) with a diagnosis of ASC as well as a non-autistic control group. In total, 198 participants took part in the study. Sixteen data sets had to be removed because participants aborted the questionnaire or filled in less than 50% of the survey items in either part of the questionnaire. The final sample included *N* = 182 participants (60 autistic participants and 122 non-autistic participants). Demographic information about the final sample is provided in Table 1, and an overview of the topics of special interests is listed in Table 2. Additional or exploratory analyses were based on the available data and are reported with the respective sample size. Categories were based on the four categories proposed by Koenig and Williams (2017) and expanded by five additional, thematically fitting categories.

**Table 1.** Demographic Information and Questionnaires.

Variable	Autistic participants *N* = 60	Non-autistic participants *N* = 122	Group statistics
Age *M* (SD)	38.7 (11.8)	38.1 (13.0)	*t*(180) = 0.27, *p* = .785
Gender			
Male	27 (45.0%)	39 (32.0%)	χ²(2) = 9.18, *p* = .010
Female	28 (46.7%)	81 (66.4%)	
Diverse	5 (8.3%)	2 (1.6%)	
Education			
Still in school	1 (1.7%)	0 (0.0%)	χ²(6) = 14.30, *p* = .026
Basic school degree (~9 years)	0 (0.0%)	0 (0.0%)	
Intermediate secondary school degree	9 (15.0%)	4 (3.3%)	
Completed vocational training	8 (13.3%)	9 (7.4%)	
Entrance qualification for universities of applied sciences	4 (6.7%)	6 (4.9%)	
General university entrance qualification (A-levels)	13 (21.7%)	29 (23.8%)	
University degree	23 (38.3%)	69 (56.6%)	
Other	2 (3.3%)	5 (4.1%)	
Employment			
Employed	31 (51.7%)	88 (72.1%)	χ²(3) = 10.73, *p* = .013
Unemployed	13 (21.7%)	10 (8.2%)	
Retired	9 (15.0%)	9 (7.4%)	
Other	7 (11.7%)	15 (12.3%)	
Autism Questionnaire *M (SD)*	39.2 (5.5)	17.5 (8.8)	*t*(179) = 17.47, *p* < .001
Liebowitz Social Anxiety Scale *M (SD)*	82.2 (27.1)	41.0 (25.3)	*t*(174) = 10.00, *p* < .001
Rosenberg Self-Esteem Scale *M (SD)*	28.0 (7.2)	32.2 (6.5)	*t*(175) = −3.89, *p* < .001

*Note.* Continuous variables are reported as *M (SD)*. Categorical variables are reported as *n (%)*, with percentages calculated within the group.

**Table 2.** Topics of (Special) Interests; Multiple Responses Were Allowed.

Category	Autistic participants	Non-autistic participants	Total	χ² (overall)
History and education	24 (40.0%)	23 (18.9%)	47 (25.8%)	χ²(8) = 52.68, *p* < .001
Creative arts	34 (56.7%)	91 (74.6%)	125 (68.7%)	
Cooking/household	3 (5.0%)	15 (12.3%)	18 (9.9%)	
Nature/animals	17 (28.3%)	24 (19.7%)	41 (22.5%)	
Travel/exploration	0 (0.0%)	25 (20.5%)	25 (13.7%)	
Science, technology, engineering, and mathematics	23 (38.3%)	19 (15.6%)	42 (23.1%)	
Sports/physical activity	11 (18.3%)	63 (51.6%)	74 (40.7%)	
TV/film/games	16 (26.7%)	23 (18.9%)	39 (21.4%)	
Other/unclear	3 (5.0%)	0 (0.0%)	3 (1.6%)	

*Note.* Values are reported as *n* (%). Percentages within groups are based on the respective group totals; percentages for the Total column are based on the full sample (*N* = 182).

Autistic participants were recruited from the Autism Outpatient Clinic at Humboldt-Universität zu Berlin. To confirm ASC diagnosis, autistic participants were asked to indicate where they had received their diagnosis. In the final sample, 85% of autistic participants reported having received their diagnosis in a specialized outpatient clinic, where ASC is routinely assessed using comprehensive clinical procedures including ADOS-2 ( Autism Diagnostic Observation Schedule, Second Edition; Lord et al., 2012) and ADI-R (Autism Diagnostic Interview–Revised; Rutter et al., 2003), a clinical interview, questionnaire-based screening, and differential-diagnostic evaluation. Fifteen percent of autistic participants indicated that they had received their diagnosis from a psychotherapist in private practice. The non-autistic group was recruited via databases and online advertising.

### Procedure

The study was approved by the local ethics committee and was presented online. Participants first provided informed consent and demographic information, as well as information on whether they had ever received an ASC diagnosis, and if so, in which facility or clinic. Before starting the first part of the survey, participants were asked to answer the questions in relation to their most important interest. The term “field of interest” was used throughout to make the survey more accessible to non-autistic participants, but we also added the information that they are referred to as special interests in ASC. Participants were then asked to list their interests, and to indicate how many hours per week they would typically engage in their interests. Subsequently, participants answered both parts of the survey; questions within both parts were presented in randomized order. Afterward, participants were given the opportunity to name additional important aspects of their interests and to provide general remarks on the questionnaire and completed the additional questionnaires (see above). The completion of the online study took approximately 30–60 min; participation was reimbursed with 12 Euro.

### Analyses

#### Part 1 – Positive Aspects of Special Interests and Negative Consequences and Stigma

In a first step, internal consistency for each of the six subscales of Part 1 of the survey was evaluated with Cronbach’s alpha. Subsequently, subscale scores were calculated by averaging the respective items for each participant. Group differences across the six subscales of Part 1 were analyzed with a linear mixed-effects model (LMM) using lmerTest (Kuznetsova et al., 2017) and the lme4 package (Bates et al., 2015) in *R* (R Core Team, 2024). The model included Group (autistic vs. control) as a between-subjects factor and Subscale (Self-Worth, Emotion Regulation, Source of Positive Emotions, Source of Learning and Knowledge, Social Identity, Negative Aspects and Stigma) as a within-subjects factor; the factor Participant was included as random intercept. Fixed effects were evaluated with omnibus *F*-tests based on the LMM, using Satterthwaite’s approximation for degrees of freedom; effect sizes were approximated with partial Cohen’s *f* (*f_p_*). Post hoc comparisons between groups were performed separately within each subscale using estimated marginal means (package emmeans; Lenth, 2025) with Bonferroni-adjustment to account for multiple comparisons. Cohen’s *d* was calculated to quantify effect sizes of significant contrasts.

In addition, reported time per week spent with the interests was compared between autistic and non-autistic participants with independent t-tests. As an additional exploratory analysis, we examined whether employment status (collapsed into employed vs. not employed) was associated with reported time spent with interests and whether it interacted with the factor Group. We therefore fitted a linear model with Group, employment status, and their interaction as predictors. This set of analyses included data of only 172 participants (54 autistic and 118 non-autistic participants), as some participants had provided verbal descriptors (e.g., “as much time as possible”) instead of reporting the exact number of hours.

In exploratory analyses, we further aimed at shedding light on negative aspects and stigma reported by our participants, to understand which factors made individuals vulnerable to experience such negative consequences. We conducted multiple regression analyses to examine the effects of autistic traits (AQ), social anxiety (LSAS), self-esteem (Rosenberg Self-Esteem Scale), time spent with interests, age, and gender on the amount of reported negative consequences and stigma. The dependent variable was quantified as the individual mean score on the Negative Aspects and Stigma subscale from Part 1 of the survey. Furthermore, interaction terms between AQ and social anxiety, self-esteem, and time spent with interests were included to explore potential moderation effects, that is, whether these variables influenced the strength of the association between autistic traits and negative consequences/stigma. The model was specified as follows: Negative Consequences and Stigma ~ AQ * LSAS *+* AQ * self-esteem *+* AQ * time spent with interest *+* *age* *+* *gender*. All continuous predictors were z-standardized prior to analysis. Multicollinearity was assessed using variance inflation factors, and residual normality was assessed using a Shapiro–Wilk test of the model residuals. Only complete cases were included in the regression (*N* = 165).

#### Part 2 – Emotion Regulation

For Part 2, responses were averaged separately for both strategies (social vs. interest) within each emotional context (positive, negative, exhausting) per participant. To examine group differences in the use of emotion regulation strategies, we employed linear mixed-effects models with Strategy (social vs. interest) and Emotional Context (positive, negative, exhausting) as within-subject factors, and Group (autistic vs. control) as a between-subject factor. The factor Participant was included as a random intercept. Post hoc analyses were conducted to investigate group differences in the general use of social vs. interest strategies, as well as interactions of these differences with emotional contexts, with Bonferroni-adjusted *p*-values to control for multiple testing.

#### Exploratory Qualitative Analysis

Participants’ responses to the optional open-ended question on additional important aspects of their interests were analyzed exploratorily using inductive category formation. In an initial analytic step, responses were grouped into preliminary thematic categories, which were then iteratively refined and labeled. The resulting categories were subsequently reviewed within the research team with regard to conceptual plausibility and the distinction between categories. This analysis was intended to provide a descriptive overview of recurring themes and to highlight potential topics for future research.

## Results

Internal consistency was in a good range for most subscales (α = 0.84–0.88) apart from the domain Social Identity, which only had acceptable internal consistency (α = .73). A linear mixed model with random intercepts for participants revealed a main effect of Group, *F*(1, 180) = 14.07, *p* < .001, *f_p_* = 0.28, and Subscale, *F*(5, 900) = 259.0, *p* < .001, *f_p_* = 1.20, as well as a significant Group × Subscale interaction, *F*(5, 900) = 10.69, *p* < .001, *f_p_* = 0.24. Post hoc analyses showed that autistic participants reported significantly higher scores than controls on the subscales Emotion Regulation, *p* = .008, *d* = 0.64, Source of Learning and Knowledge, *p* = .014, *d* = 0.61, and Negative Aspects and Stigma, *p* < .001, *d* = 1.39. No significant group differences were observed for Self-Worth, Social Identity, or Source of Positive Emotions. In sum, the magnitude of group differences varied across subdomains, with the largest effect observed for Negative Aspects and Stigma. Mean scores and distributions of all subscales are depicted in Figure 1.

**Figure 1.**
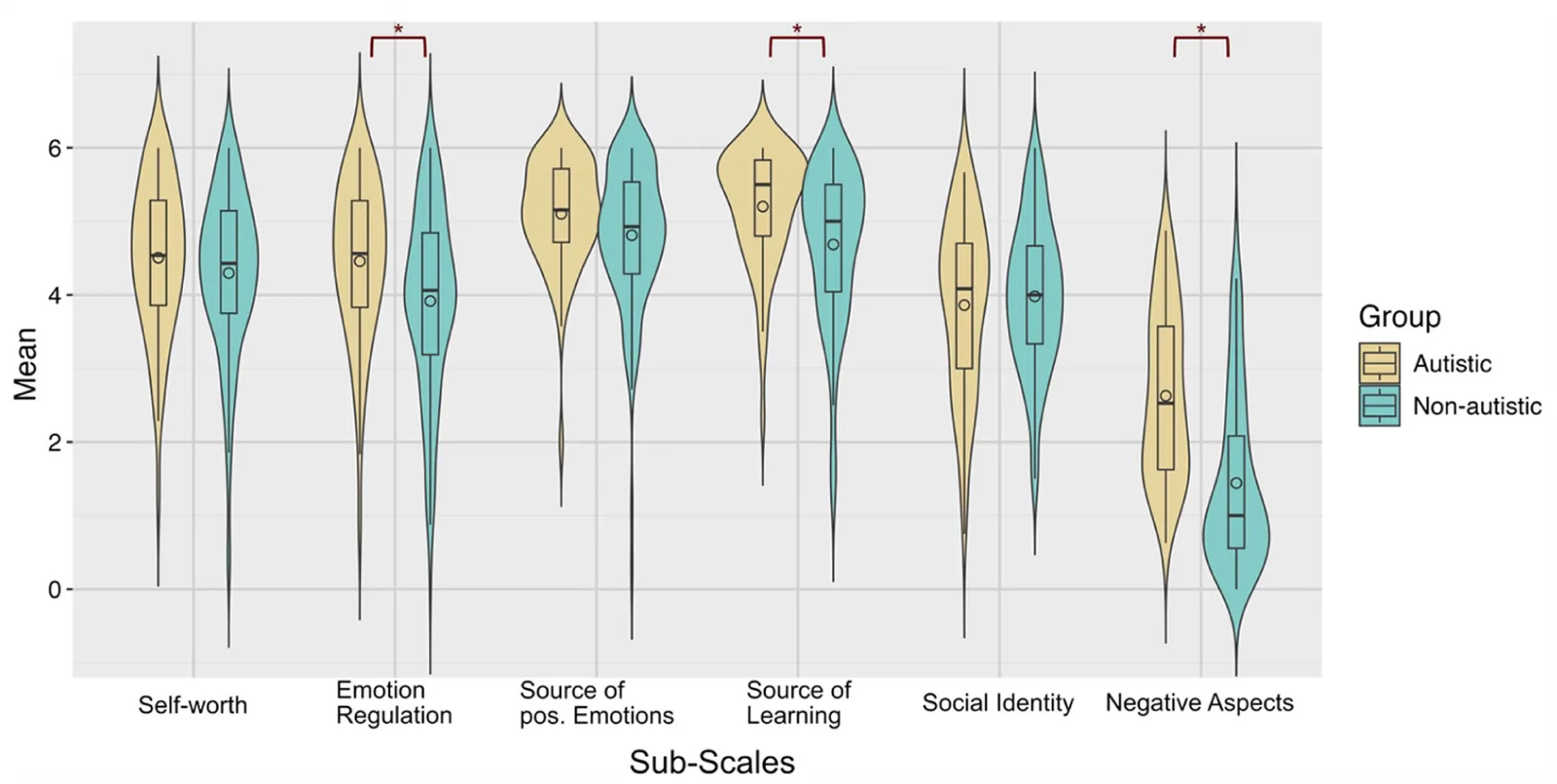
Mean observed scores across subscales for autistic and non-autistic participants. Scores range from 0 to 6. Violin plots represent the distribution of scores per group; overlaid boxplots show the interquartile range and median; mean values are marked with circles. Please note that violin plots are smoothed density distributions and may therefore extend slightly beyond the observed scale limits.

Analyses further revealed that autistic participants spent significantly more time with their interests (*M* = 28.6 h, *SD* = 19.6 h) than non-autistic participants (*M* = 14.2 h, *SD* = 14.4 h), *t*(170) = 5.4, *p* < .001. In additional analyses that included employment status (collapsed into employed vs. not employed), the effect of Group remained significant, *F*(1, 168) = 29.19, *p* < .001, whereas neither employment status, *F*(1, 168) = 0.08, *p* = .772, nor the Group × Employment interaction, *F*(1, 168) = 1.89, *p* = .171, were significant.

In exploratory analyses, we used multiple regression to investigate which factors were associated with the extent of negative consequences and stigma reported by participants, and whether social anxiety, self-esteem, and time spent with interests moderated the association between autistic traits and negative consequences/stigma. The overall model was significant, *F*(10, 154) = 17.48, *p* < .001, explaining 53.2% of the variance in the dependent variable (adjusted *R*² = .50). AQ emerged as a significant positive predictor of negative consequences and stigma, *B* = 0.56, *SE* = 0.11, *t*(154) = 5.28, *p* < .001. Further significant positive associations were found for time spent with interests, *B* = 0.22, *SE* = 0.09, *t*(154) = 2.35, *p* = .020, and social anxiety, *B* = 0.34, *SE* = 0.13, *t*(154) = 2.71, *p* = .008, whereas age showed a significant negative association, *B* = −0.19, *SE* = 0.08, *t*(154) = −2.55, *p* = .012. Self-esteem and gender were not significant predictors. No significant moderation effects were observed for social anxiety, self-esteem, or time spent with interests, all *p*s > .11. Thus, social anxiety, self-esteem, and time spent with interests did not significantly moderate the association between autistic traits and negative consequences and stigma.

In Part 2 of the questionnaire, we compared the use of social strategies and special interests as a means of emotion regulation separately for positive, negative, and exhausting situations. A linear mixed model with Group, Strategy, and Emotional Context as fixed effects and Participant as a random intercept revealed significant main effects of Group, *F*(1, 180) = 7.35, *p* = .007, *f*_p_ = 0.20, Strategy, *F*(1, 900) = 247.26, *p* < .001, *f*_p_ = 0.52, and Emotional Context, *F*(2, 900) = 187.01, *p* < .001, *f*_p_ = 0.64. Importantly, the interaction between Group and Strategy was significant, *F*(1, 900) = 184.56, *p* < .001, *f*_p_ = 0.45; reflecting the finding that autistic participants, across emotional contexts, reported significantly lower use of social strategies compared with controls, *p* < .001, *d* = −1.20, but higher use of interest-based strategies, *p* < .001, *d* = 0.55. Moreover, the three-way interaction Group × Strategy × Emotional Context also reached significance, *F*(2, 900) = 4.76, *p* = .009, *f*_p_ = 0.10. Bonferroni-corrected post hoc tests revealed that group differences were most pronounced in negative and exhausting contexts: autistic participants were less likely to use social strategies than non-autistic participants (Negative: *p* < .001, *d* = −1.47; Exhausting: *p* < .001, *d* = −0.91), but reported higher use of interest strategies (Negative: *p* < .001, *d* = 0.83; Exhausting: *p* = .028, *d* = 0.46). In positive contexts, autistic participants were also less likely to seek social contact, *p* < .001, *d* = −1.23. However, in positive contexts, there was no significant difference between groups in engagement in interests, *p* = .125, *d* = 0.35. Results are summarized in Figure 2.

**Figure 2.**
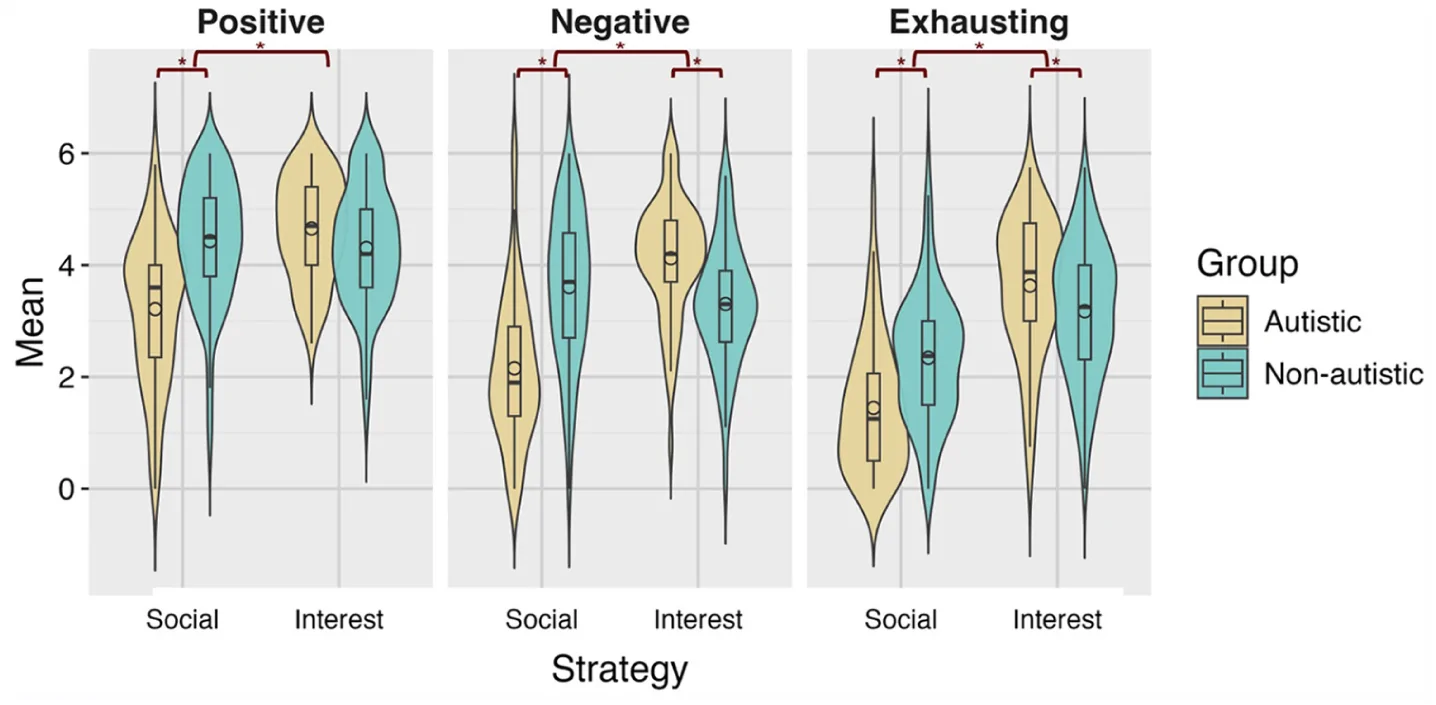
Mean observed scores for the likelihood of social and interest-based strategies in positive, negative, and exhausting situations, shown separately for autistic and non-autistic participants. Scores range from 0 to 6. Violin plots represent the distribution of scores per group; overlaid boxplots show the interquartile range and median; mean values are marked with circles. Note that the violin plots are smoothed density distributions and may therefore extend slightly beyond the observed scale limits.

### Exploratory Qualitative Analyses

A total of 80 participants provided optional open-ended responses (33 autistic and 47 non-autistic). The most frequently recurring themes concerned positive functions and meanings of interests, particularly their links to positive emotions, balance in everyday life, and psychological and physical well-being. They were also often described as an important part of personal identity and development. A second recurring theme concerned the connection between interests and education or work. Participants noted that their interests could influence educational and career choices, support professional development, or be directly integrated into study or work contexts. A third, less frequently mentioned theme concerned the social role of interests, and how they could facilitate social contact, provide shared topics or activities, and in some cases contribute to the development of social skills. Finally, some participants also described negative aspects associated with their interests, including pressure, compulsive tendencies, frustration, and stress, particularly when the interest was experienced as difficult to disengage from or tied to high personal standards.

## Discussion

Our findings highlight the importance of special interests for emotional and psychological well-being, particularly among autistic individuals. Autistic participants reported a significantly higher impact of their special interests on the dimensions of emotion regulation and source of learning and knowledge compared with the control group, while also experiencing greater stigma and negative consequences related to their interests. However, it is noteworthy that interests were also perceived as highly meaningful by control participants, underlining their broader relevance for personal well-being. The second part of the survey further emphasized the regulatory function of special interests for autistic individuals, who reported a stronger tendency to rely on their interests as a coping strategy in negative and exhausting situations compared with non-autistic participants.

Our findings highlight the importance and relevance of special interests for autistic individuals as a source of well-being. This is in line with previous studies that incorporated the autistic perspective on special interests instead of taking more deficit-oriented viewpoints (Grove et al., 2018; Koenig & Williams, 2017; Mercier et al., 2000; Winter-Messiers, 2007). More broadly, the present findings also align with recent flourishing-oriented perspectives in autism research, which argue for a broader understanding of autistic well-being than mental health alone (Pellicano & Heyworth, 2023). In this context, meaningful engagement, supportive environments, and opportunities to build on personal strengths and valued interests have been highlighted as important aspects of well-being and fulfillment (Davies et al., 2025). At the same time, considering parental alongside autistic perspectives can further enrich our understanding of lived experiences and support needs (Hoffman & Kirby, 2022).

Our findings are also in line with recent neuroimaging evidence showing greater activation of the brain’s reward system – specifically, increased responses of the caudate to special interests compared with social stimuli in autistic individuals (Kohls et al., 2018). In addition, autistic individuals showed increased amplitudes of the late positive potential (LPP) (Benning et al., 2016), a higher-order ERP component associated with sustained processing of emotionally and motivationally significant stimuli (Hajcak & Foti, 2020). Moreover, they assigned higher valence ratings to special interest stimuli than to social stimuli (Sasson et al., 2012), suggesting a more positive evaluation. Due to the rewarding and motivating nature of special interests in ASC (Grove et al., 2016, 2018), they have also been successfully implemented in interventions for children to foster adaptive behavior and social communication (see Harrop et al., 2019). Furthermore, current NICE guidelines for autistic adults recommend integrating special interests into interventions targeting co-occurring mental health conditions, as a means of enhancing engagement and sustaining attention (National Institute for Health and Care Excellence, 2021). More broadly, recent work has emphasized that autistic interests and strengths may be harnessed in interventions to promote well-being, learning, and adaptive functioning (Courchesne et al., 2020).

Beyond their motivational significance, our findings highlight the important role of special interests in emotion regulation. In the first part of our survey, autistic individuals reported a higher importance of special interests for regulating negative emotions compared with the control group. A closer examination of emotion regulation in the second part of our survey revealed that autistic individuals, compared with the control group, were more likely to engage with their interests during negative and exhausting situations, thereby demonstrating their potential as a coping mechanism to alleviate stress. Given the substantial psychosocial challenges that autistic individuals may encounter, as well as the high prevalence of co-occurring mental health conditions, the development and implementation of effective strategies for preventing and managing stress is of considerable clinical relevance. Existing cognitive-behavioral therapy (CBT) manuals (e.g., Dziobek & Stoll, 2019) already emphasize the importance of accepting autistic traits and promoting individualized, autism-friendly spaces, i.e., environments in which autistic individuals can be their authentic selves and act according to their preferences and needs, for example, by engaging in their interests or adhering to routines.

Our findings suggest that engagement in interests may offer benefits beyond these established aspects. Specifically, engagement in interests appears to function as an active coping strategy for managing stressful situations and regulating negative emotions. This interpretation aligns with and extends previous work on emotion regulation in autism: Cai and colleagues (2018) emphasized that autistic individuals may show distinct emotion-regulation patterns, sometimes involving lower flexibility and a narrower range of available strategies compared with non-autistic individuals. They called for research to identify context-dependent and autism-relevant forms of emotion regulation. In this light, our findings suggest that engaging in special interests may constitute such a contextually adaptive and self-relevant strategy, supporting emotional stability. While the potential of special interests to serve as an emotion regulation strategy in ASC had been identified by previous research (e.g., Koenig & Williams, 2017; Lizon et al., 2024), our study was the first one to compare the use of interests with social strategies and to distinguish situational characteristics in a within-subject design. Our findings indicate distinct patterns of coping between autistic and non-autistic individuals that vary according to situational context: Notably, in positive situations, both groups reported comparable engagement with their interests, even though autistic individuals were significantly less likely to seek social contact. In contrast, in negative situations, autistic individuals reported a stronger reliance on their interests and a reduced tendency to employ social strategies compared with non-autistic participants. Thus, group differences were most pronounced under adverse conditions, with potential implications for psychotherapy: First, the findings support encouraging autistic individuals to actively use and cultivate their special interests as an adaptive coping resource. Second, if strengthening social bonds is identified as a therapeutic goal, our data suggest that positive contexts may offer the most favorable conditions for engaging in social interaction, as individuals are more likely to have sufficient resources available to tolerate its demands, especially given that social contacts themselves seem to constitute a source of stress for many autistic individuals (Black et al., 2023). In contrast, strategies aimed at strengthening the use of social support during periods of acute stress, which have been linked to beneficial mental health effects in non-autistic populations (Li et al., 2021), may be less helpful for autistic individuals.

Furthermore, it seems noteworthy that, contrary to our hypothesis, no significant group differences emerged regarding the role of interests for self-worth, as a source of positive emotions, or social identity in Part 1 of our survey. These findings highlight the fact that interests (usually referred to as hobbies in research with non-autistic individuals) are of substantial importance not only for autistic individuals, but also for the general population. A recent review identified three domains consistently associated with hobby engagement: lower depression, anxiety, and stress; better quality of life and well-being; and stronger social interaction and support (Cleary et al., 2025). This broader pattern is in line with findings from Mak et al. (2023), who reported that hobby engagement was associated with fewer depressive symptoms, greater life satisfaction, higher happiness, and better self-reported health in a large sample of adults aged 65 years and older. Future research should examine more directly how interests relate to mental health, quality of life, life satisfaction, and support-related processes in autistic and non-autistic adults, as interests may be broadly beneficial across groups while still differing in the specific roles they play. This point is also relevant when considering the demographic differences between groups in the present study, particularly with regard to gender, given the higher proportion of women in the control group than in the ASC group, but also the relatively high number of women in the ASC group. Such differences may affect the generalizability of the findings and may also contribute to variation in the specific roles interests play, especially given evidence that gender differences may influence the content or intensity of interests in individuals with ASC (Bourson & Prevost, 2024; Edwards et al., 2024). Likewise, gender differences have been reported in non-autistic populations with regard to the content of interests (Su et al., 2009), and more generally in the use of coping strategies (Graves et al., 2021) and stigma perception (Pedersen & Paves, 2014), which may also have contributed to the pattern of findings observed here. In addition, our groups differed in employment status, with higher unemployment rates among autistic participants, which is in line with evidence that autistic adults continue to face substantial barriers in education and especially in employment (Bury et al., 2024). At the same time, the autistic sample in the present study also included a relatively high proportion of university students or graduates, and more than half of the autistic participants were employed. These characteristics should be taken into account when considering the representativeness of the sample and the generalizability of the findings. However, in our additional analysis investigating the time spent with interests, the group difference remained significant when employment status was included, and employment itself did not have a significant effect on the time spent with the interests. At the same time, our exploratory regression analyses should be interpreted cautiously, given their limited statistical power in the present sample, and future research should therefore examine these questions in larger samples.

While engagement in interests seems to be associated with numerous benefits, our data also shed light on potential negative aspects: autistic participants reported significantly higher levels of negative consequences and perceived stigma associated with their interests. This is in line with previous work showing that, although special interests are often linked to positive outcomes, a high intensity of engagement may also be associated with lower well-being in autistic adults (Grove et al., 2018). In our exploratory regression analyses across groups, higher levels of negative consequences and stigma were associated with higher autistic traits, higher social anxiety, more time spent with interests, and younger age. Interestingly, no moderation effects of social anxiety, time spent with interests, or self-esteem on the association between autistic traits and negative consequences were observed. Therefore, interventions targeting the reduction of social anxiety may be a promising avenue to mitigate the negative consequences of engaging in interests across both autistic and non-autistic individuals, especially given the heightened vulnerability to negative evaluation associated with social anxiety (Heimberg et al., 2014). The association with younger age may suggest greater vulnerability to stigma-related concerns in younger adults, consistent with findings that stigma levels tend to be lower in older adults (Mackenzie et al., 2019). At the same time, our findings suggest that additional factors may contribute to the substantially higher levels of negative consequences and stigma reported by autistic individuals. For instance, difficulties may arise from the style of communicating about special interests, such as when autistic individuals may be unaware of whether the interest is shared or reciprocal. From a clinical perspective, this may suggest that interventions should help autistic individuals develop strategies for communicating about their interests in ways that reduce misunderstanding and negative social consequences. More broadly, acceptance- and commitment-based approaches may represent a promising avenue to reduce stress and increase quality of life (Pahnke et al., 2023).

Additional negative aspects were also mentioned in the open-ended responses, particularly burdens more directly linked to engagement in the interest itself, such as pressure, frustration, or difficulty disengaging. These aspects fit well with the passion literature, which distinguishes between harmonious and obsessive forms of engagement in meaningful activities, depending on whether the activity is integrated into a person’s life in a more flexible and balanced way or experienced as more rigid and difficult to regulate (Vallerand et al., 2003) and suggests that these may have different implications for well-being and psychological burden (Vallerand, 2012). This interpretation is also broadly consistent with autism research indicating that the way restricted interests are expressed may be linked to anxiety, at least in individuals with co-occurring anxiety symptoms (Spiker et al., 2012). Future research should therefore examine more directly how different forms of engagement with interests relate to well-being and psychological burden in autistic adults.

Several limitations should be considered when interpreting the present findings. A first limitation concerns the sample characteristics and the comparability of the groups. Although autistic and non-autistic participants were similar in age, they differed in gender distribution as well as in education and employment status, which may limit the generalizability of the findings and may have influenced outcomes. Moreover, the sample size limited the statistical power of the exploratory regression analyses, especially for interaction effects, and these findings should therefore be interpreted cautiously. Regarding the subscales of the first part of the questionnaire, it should be taken into account that the negative subscale was broader than the positive subscales and combined multiple aspects; furthermore, the Social Identity subscale showed only acceptable internal consistency and may therefore be revised in future research.

A further limitation is that the diagnostic status of autistic participants was not independently verified, but relied on self-report, although all autistic participants reported the setting in which they had received their diagnosis. Responses may also have been influenced by the online format and by social desirability or other self-report biases. Furthermore, the study did not distinguish between different modes of engagement with interests, such as more harmonious versus more obsessive forms, which may be relevant for understanding why interests are associated with both beneficial and burdensome experiences. Future studies should therefore use larger and more demographically balanced samples and examine these different forms of engagement more directly, while also differentiating more clearly between different types of negative aspects, to better understand how engagement with interests relates to well-being across individuals.

In conclusion, our findings highlight the important role of special interests for autistic individuals, both as a source of well-being and as a strategy for coping with negative emotions. At the same time, engaging in special interests was associated with stigma and negative consequences, especially for autistic individuals, calling for therapeutic strategies to alleviate the negative aspects of engagement in special interests, while using their full potential for increasing mental health and well-being.

## Supplemental Material

sj-docx-1-aut-10.1177_13623613261471572 – Supplemental material for Special Interests in Autism: Functions, Benefits, and Challenges Supplemental material, sj-docx-1-aut-10.1177_13623613261471572 for Special Interests in Autism: Functions, Benefits, and Challenges by Mareike Bayer and Isabel Dziobek in Autism

## Data Availability

Data are not publicly available as the detailed and highly specific personal information about participants’ special interests could compromise their anonymity. Code is available at https://osf.io/t7wcf/overview?view_only=426c6193cc354f9e84939246882661e7.

## Notes

1. We will use the term special interests for the sake of consistency; in the online survey, the expression field of interest was used, see below.

2.
https://www.autismus-forschungs-kooperation.de
