# Supplementary material for: Special Interests in Autism: Functions, Benefits, and Challenges
Source: Autism. 2026 Aug 6;30(10):2545–57. doi: 10.1177/13623613261471572 (PMC13624314; doi:10.1177/13623613261471572)
Supplement: Supplementary file 1 [file sj-docx-1-aut-10.1177_13623613261471572.docx]

**Supplementary Statistical Results**

# Part 1: Positive Aspects and Negative Consequences/Stigma

Sample size: N = 182 (60 autistic; 122 non-autistic).

Table S1: Omnibus tests from the linear mixed-effects model.

| **Effect** | **df1** | **df2** | **F** | **p** | **fᵖ** |
| --- | --- | --- | --- | --- | --- |
| Group | 1 | 180 | 14.07 | < .001 | .28 |
| Subscale | 5 | 900 | 258.95 | < .001 | 1.20 |
| Group × Subscale | 5 | 900 | 10.69 | < .001 | .24 |

Table S2: Bonferroni-adjusted group contrasts within each subscale.

| **Subscale** | **Estimate** | **SE** | **df** | **t** | **p** | **d [95% CI]** |
| --- | --- | --- | --- | --- | --- | --- |
| Self-worth | .21 | .17 | 641 | 1.23 | .220 | .24 [-.15, .63] |
| Emotion regulation | .54 | .17 | 641 | 3.21 | .001 | .64 [.25, 1.03] |
| Source of pos. emotions | .29 | .17 | 641 | 1.72 | .085 | .34 [-.05, .73] |
| Source of learning | .52 | .17 | 641 | 3.05 | .002 | .61 [.21, 1.00] |
| Social identity | -.12 | .17 | 641 | -.71 | .480 | -.14 [-.53, .25] |
| Negative aspects | 1.19 | .17 | 641 | 7.02 | < .001 | 1.39 [1.00, 1.79] |

# Time Spent with Interests

Sample size for the time analyses: N = 172 (54 autistic; 118 non-autistic).

Table S3: Group comparison for time spent with interests.

| **Analysis** | **Statistic** | **p** | **Effect size** |
| --- | --- | --- | --- |
| Group comparison | t(170) = 5.40 | < .001 | d = .89 |

**Extended linear model including employment status and the Group × Employment interaction: F(3, 168) = 12.40, p < .001, adjusted R² = .17.**

Table S4: Group comparison for time spent with interests, accounting for employment status.

| **Effect** | **df1** | **df2** | **F** | **p** | **η²p** |
| --- | --- | --- | --- | --- | --- |
| Group | 1 | 168 | 29.19 | < .001 | .15 |
| Employment status | 1 | 168 | 0.08 | .772 | .00 |
| Group × Employment status | 1 | 168 | 1.89 | .171 | .01 |

# Part 2: Emotion Regulation Strategies

Table S5: Omnibus tests from the linear mixed-effects model.

| **Effect** | **df1** | **df2** | **F** | **p** | **η²p** | **fᵖ** |
| --- | --- | --- | --- | --- | --- | --- |
| Group | 1 | 180 | 7.35 | .007 | .039 | .20 |
| Strategy | 1 | 900 | 247.26 | < .001 | .216 | .52 |
| Emotional Context | 2 | 900 | 187.01 | < .001 | .294 | .64 |
| Group × Strategy | 1 | 900 | 184.56 | < .001 | .170 | .45 |
| Group × Emotional Context | 2 | 900 | .97 | .380 | .002 | .05 |
| Strategy × Emotional Context | 2 | 900 | 16.58 | < .001 | .036 | .19 |
| Group × Strategy × Emotional Context | 2 | 900 | 4.76 | .009 | .010 | .10 |

Table S6: Bonferroni-adjusted group contrasts.

| **Context** | **Strategy** | **Estimate** | **SE** | **df** | **t** | **p** | **d [95% CI]** |
| --- | --- | --- | --- | --- | --- | --- | --- |
| Across contexts | Social | -1.19 | .14 | 292 | -8.79 | < .001 | -1.20 [-1.48,  -.93] |
| Across contexts | Interest | .54 | .14 | 292 | 4.00 | < .001 | .55 [.28, .82] |
| Positive | Social | -1.22 | .19 | 752 | -6.55 | < .001 | -1.23 [-1.60,  -.86] |
| Positive | Interest | .35 | .19 | 752 | 1.86 | .063 | .35 [-.02, .72] |
| Negative | Social | -1.45 | .19 | 752 | -7.82 | < .001 | -1.47 [-1.84,  -1.09] |
| Negative | Interest | .82 | .19 | 752 | 4.41 | < .001 | .83 [.46, 1.20] |
| Exhausting | Social | -.89 | .19 | 752 | -4.82 | < .001 | -.91 [-1.28, -.53] |
| Exhausting | Interest | .46 | .19 | 752 | 2.47 | .014 | .46 [.09, .83] |

# Exploratory Regression Analysis

Sample size: N = 165.

Overall model fit: F(10, 154) = 17.48, p < .001, R² = .532, adjusted R² = .501.

Table S7: Exploratory regression analyses

| **Predictor** | **Estimate** | **SE** | **t** | **p** |
| --- | --- | --- | --- | --- |
| Intercept | 1.862 | .119 | 15.65 | < .001 |
| AQ (z) | .557 | .105 | 5.28 | < .001 |
| Time spent with interests (z) | .217 | .092 | 2.35 | .020 |
| Self-esteem (z) | .017 | .098 | .17 | .863 |
| Social anxiety (z) | .344 | .127 | 2.71 | .008 |
| Age (z) | -.194 | .076 | -2.55 | .012 |
| Gender: male | .028 | .161 | .17 | .864 |
| Gender: diverse | .274 | .405 | .68 | .499 |
| AQ × Time | -.135 | .086 | -1.58 | .117 |
| AQ × Self-esteem | -.106 | .090 | -1.18 | .240 |
| AQ × Social anxiety | -.018 | .109 | -.17 | .867 |

Assumption checks indicated no problematic multicollinearity. Shapiro–Wilk test suggested a slight deviation from residual normality (W = 0.98, p = .040).

| **Assumption check** | **Statistic** | **p / Value** |
| --- | --- | --- |
| Residual normality | Shapiro–Wilk W = 0.9 | p = .040 |
| Multicollinearity | GVIF range | 1.14-3.21 |
